# Characterization of conformational heterogeneity via higher-dimensionality, proton-detected solid-state NMR

**DOI:** 10.1007/s10858-022-00405-0

**Published:** 2022-09-23

**Authors:** Ekaterina Burakova, Suresh K. Vasa, Rasmus Linser

**Affiliations:** 1 Department of Chemistry and Chemical Biology, Technical University Dortmund, Otto-Hahn-Str. 4a, 44227 Dortmund, Germany; 2 Department of Chemistry and Pharmacy, Ludwig-Maximilians-University Munich, Butenandtstr. 5-13, 81377 Munich, Germany

**Keywords:** Fast-MAS solid-state NMR, Proton detection, Sample heterogeneity, TALOS, PACSY

## Abstract

Site-specific heterogeneity of solid protein samples can be exploited as valuable information to answer biological questions ranging from thermodynamic properties determining fibril formation to protein folding and conformational stability upon stress. In particular, for proteins of increasing molecular weight, however, site-resolved assessment without residue-specific labeling is challenging using established methodology, which tends to rely on carbon-detected 2D correlations. Here we develop purely chemical-shift-based approaches for assessment of relative conformational heterogeneity that allows identification of each residue via four chemical-shift dimensions. High dimensionality diminishes the probability of peak overlap in the presence of multiple, heterogeneously broadened resonances. Utilizing backbone dihedral-angle reconstruction from individual contributions to the peak shape either via suitably adapted prediction routines or direct association with a relational database, the methods may in future studies afford assessment of site-specific heterogeneity of proteins without site-specific labeling.

**Supplementary Information:**

The online version contains supplementary material available at 10.1007/s10858-022-00405-0.

## Introduction

Protein disorder plays a significant role in various cellular processes (Uversky 2013, 2018). Intrinsically disordered proteins (IDPs) and intrinsically disordered protein regions (IDRs), due to their high flexibility and high accessibility, are crucial elements of transcription factors (Sammak and Zinzalla 2015), voltage-dependent gating (Zhou et al. 2001; Kjaergaard and Kragelund 2017), protein phase separation (Turoverov et al. 2019), and many others. Intrinsic disorder gives proteins the ability to form low-affinity but highly specific complexes, which is important for regulatory pathways (Uversky 2013). Similarly, ensembles of partially folded intermediates can provide valuable insight into protein folding, mis- or refolding (Havlin and Tycko 2005a; Hu et al. 2009; Potapov et al. 2015). Whereas NMR has proven to be an invaluable tool to characterize the level of disorder in solution (Lindorff-Larsen et al. 2004; Nielsen and Mulder 2020), static disorder in the solid state, either related to the conformational ensemble of the protein in solution or as a biological property on its own, can be assessed by solid-state NMR spectroscopy (Siemer 2020). Upon aggregation, solidification, or even crystallization, disorder can be captured for part of the protein sequence, even if other parts transition into well-ordered structural elements. As a consequence, heterogeneity in the solid state is of significance for example for understanding the aggregation mechanisms of amyloids (Morris et al. 2012; Elkins et al. 2016; Xiang et al. 2017) as well as the formation principles of complex biological conjugates such as spider silk (Asakura et al. 2013a, b). “Arrested dynamic disorder” can be quantified in freeze-trapped solutions (Havlin and Tycko 2005b; Heise et al. 2005), which can capture the previous physiologically relevant conformational distribution. Disorder in the solid state may manifest itself in the presence of a low number of distinct forms, as it is sometimes the case for proteins in polymorphic amyloid assemblies (Paravastu et al. 2008; Tycko 2011, 2014; Amo et al. 2012; Jaroniec 2019). Alternatively, a distribution of numerous conformations can arise, as sometimes found for membrane protein preparations already at room temperature (Su and Hong 2011), for crystalline proteins upon freezing (Luo and Yu 2008; Linden et al. 2011; Siemer et al. 2012; Endapally et al. 2019), for folding intermediates (Chimon and Ishii 2005; Havlin and Tycko 2005b), or freeze-trapped IDRs (Hu and Tycko 2010), which in the extreme case can span a continuously sampled conformational space. With significant recent improvements in hardware, there is a growing interest in utilization of solid-state NMR assessment not only using standard MAS approaches but also—given the intrinsically low sensitivity of frozen solutions and the general hurdle of broad (and thus lower-height) peaks—via dynamic nuclear polarization (DNP) techniques (Siemer et al. 2012; Uluca et al. 2018; Jeon et al. 2019).

Different approaches toward quantitative characterization of the static disorder have been described, where either isotropic chemical shifts, CSA, or dipolar coupling correlations are used and translated into best-fit ensemble models via principle-component analysis of spectral features or via MD-derived ensembles *a-posteriori* weighted by comparison of in-silico with experimental data. Despite the enormous challenges on the way, e. g., the faithful simulation of isotropic chemical shifts of individual conformer contributions, the often-underdetermined relationship between experimental values and dihedral properties, as well as the resolution limitations occurring for the desired type of experiment for a more complex target protein, quantitative insights into secondary-structure polymorphism have been obtained in several studies by deconvolution of ^13^C-detected NMR spectra. Some examples are an analysis of measured and simulated peaks in static 2D spin-diffusion experiments in model polymers (Asakura et al. 2001) or spider silk with isotope labeling by residue type (Kümmerlen et al. 1996), assessment of spider silk via DOQSY spectra (Beek et al. 2000), the conformation of peptide T in frozen glycerol/water solution using 2D ^13^C–^13^C exchange spectroscopy (Dios et al. 2004), double quantum/zero quantum- (DQ–ZQ) spectroscopy of the neurotensin peptide without cryoprotectant (Heise et al. 2005), a linear combination of “clean” SQ/SQ spectra or correlated anisotropic interactions of carbonyls for assessing the unfolding of HP35 (Havlin and Tycko 2005a; Hu et al. 2009; Hu and Tycko 2010), PCA analysis of melittin spectra interrupted upon folding (Hu et al. Oct. 2009), selectively labeled α-synuclein in frozen solution (Uluca et al. Apr. 2018), and others. One of the biggest hurdles in the presence of conformational ensembles is sufficient signal dispersion for downstream processing of NMR data for more in-depth analyses. To overcome it, selective amino acid labeling is often used (Tycko 2014), which, however, largely reduces the information content per sample and necessitates a high reproducibility of the sample preparation in case multiple sites are to be investigated.

**Fig. 1:**
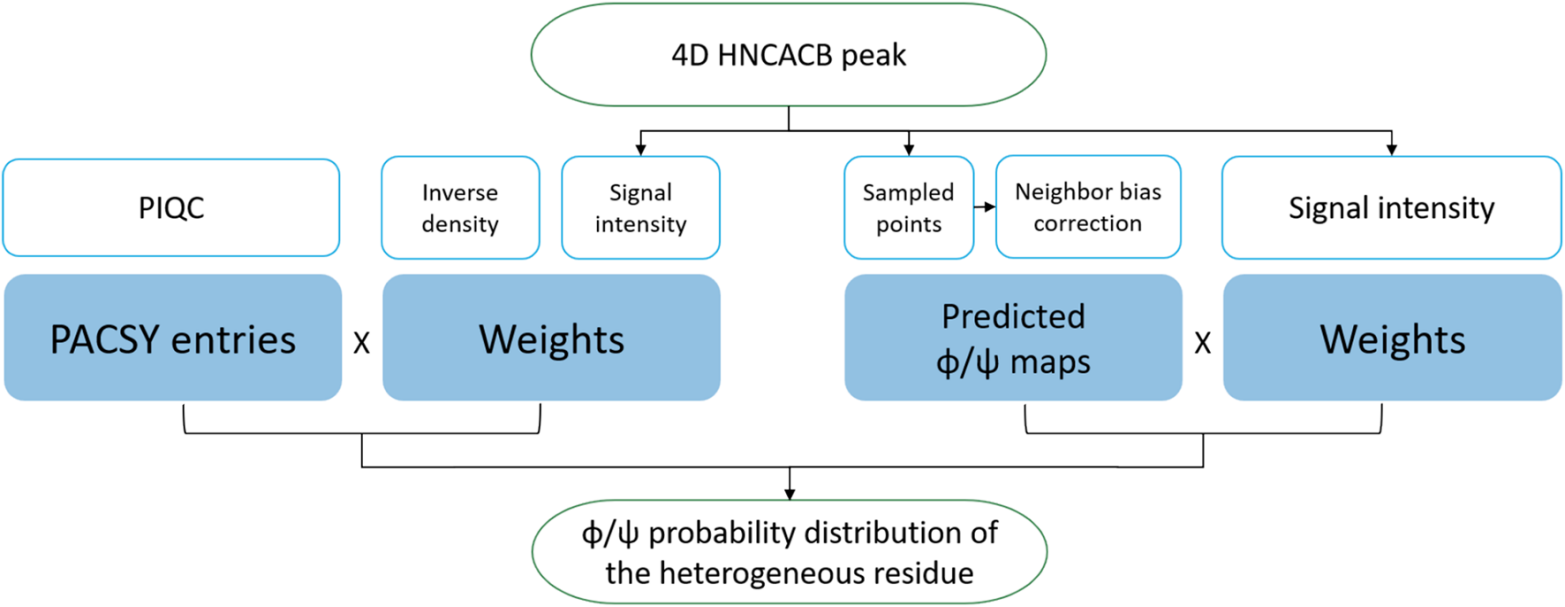
Flowchart of the two developed approaches to study peak shapes in higher-dimensionality solid-state NMR spectra of heterogeneous proteins via chemical-shift patterns. Left: PACSY data base-derived reconstruction of conformational ensembles, right: prediction-based assessment of backbone dihedral-angle distributions

In this work, to enable the readout of multiple heterogeneously broadened peaks (from different residues) at once, we explore two purely chemical-shift-based approaches for residue-specific evaluation of the dihedral angle distribution in conjunction with higher-dimensionality spectra. The first one relies on exploiting chemical-shift based dihedral-angle predictions, most importantly via TALOS-N (Shen and Bax 2013). A complementary approach is a direct chemical shift database comparison (Fig. 1). For a possible utilization for future biological questions, we specifically include chemical shifts of backbone H^N^, N^H^, and C^α^ nuclei as well as C^β^ (i.e. four dimensions) to achieve a chemical shift dispersion as large as possible, which gives access to peak features within the higher-dimensionality shift correlations. To be able to interpret the resulting conformational ensembles in terms of a degree of conformational heterogeneity, we suggest a series of scores, which we apply to the outcomes of both reconstruction frameworks.

## Materials and methods

Uniformly labelled (^13^C, ^15^ N)-GGAGG pentapeptide was purchased from Thermo Fischer. An inhomogeneous sample was prepared by dissolving the peptide in 1 mL of ddH_2_O, flash-freezing in liquid nitrogen, and drying in a vacuum chamber at 0.01 bar. Spectra of GGAGG were recorded on a Bruker Avance 800 MHz NMR spectrometer in a 1.3 mm MAS rotor. The rotor was filled by overnight centrifugation of the sample and spun at 40 kHz at a temperature of 10 °C. A 4D hCBCANH spectrum (Xiang et al. 2016) of GGAGG was acquired non-uniformly with 5% sampling density (19,208 points). A Poisson-Gap schedule and hmsIST as the reconstruction method were chosen based on previous work (Burakova et al. 2020). The DREAM (Verel et al. 2001) scheme was used to achieve C^α^–C^β^ magnetization transfer. Digital resolution in the indirect dimensions was 127.7 Hz for nitrogen and 251.0 Hz in both carbon dimensions. The spectrum was recorded in seven blocks of eight scans to allow for manual field correction in between. Each block was recorded for ca. 2.5 days. The 2D correlation spectra were referenced to an external DSS standard as described in Aeschbacher et al. (2012). The 4D hCBCANH was indirectly referenced by superimposing the 2D projections with the 2D DREAM correlation. For details on acquisition and processing parameters see Table S1. Apodization had no significant effect on the GGAGG peak shapes and widths in comparison to the inhomogeneous broadening (see Fig. S1). Spectral processing was done using NMRPipe software (Delaglio et al. 1995).

The CSV-formatted PACSY database (version from Dec, 28 2020) (Lee et al. 2012), cleansed by methodology presented in Fritzsching et al. (2016), was analyzed and visualized using Python and Python-based packages including NumPy (Harris et al. 2020), NMRglue (Helmus and Jaroniec 2013), Pandas (McKinney et al. 2010) and others (Hunter 2007; Waskom et al. 2017; Fundamental Algorithms for Scientific Computing in Python et al. 2020).

## Results

### Model heterogeneous sample

We developed our approach on a short model heterogeneous sample of u-(^13^C, ^15^ N)-GGAGG pentapeptide, checked for purity by mass spectrometry and analytical HPLC. We introduced a permanent conformational disorder by first flash-freezing in water and then freeze-drying to 10 mbar, resulting in a glass. Being minimally restrained by steric properties, the G-A-G peptide bonds in this sample can be assumed to represent an extreme example of dihedral-angle variability. In this sample, peak broadening can be assumed to derive almost exclusively from conformational heterogeneity, as long-range modulation of the chemical shift by ring-current effects were specifically avoided. Contributions from intermolecular contacts to the heterogeneous line shape, beyond the backbone dihedral angles of interest, can be assumed to be largely limited to the H and N shifts (due to differential H-bonding interactions). These are an acceptable compromise for developing the demonstrated algorithms, as in future applications in frozen solutions these would be severely reduced and because carbon shifts are less susceptible to intermolecular contacts. (See a discussion of this limitation below.)

**Fig. 2:**
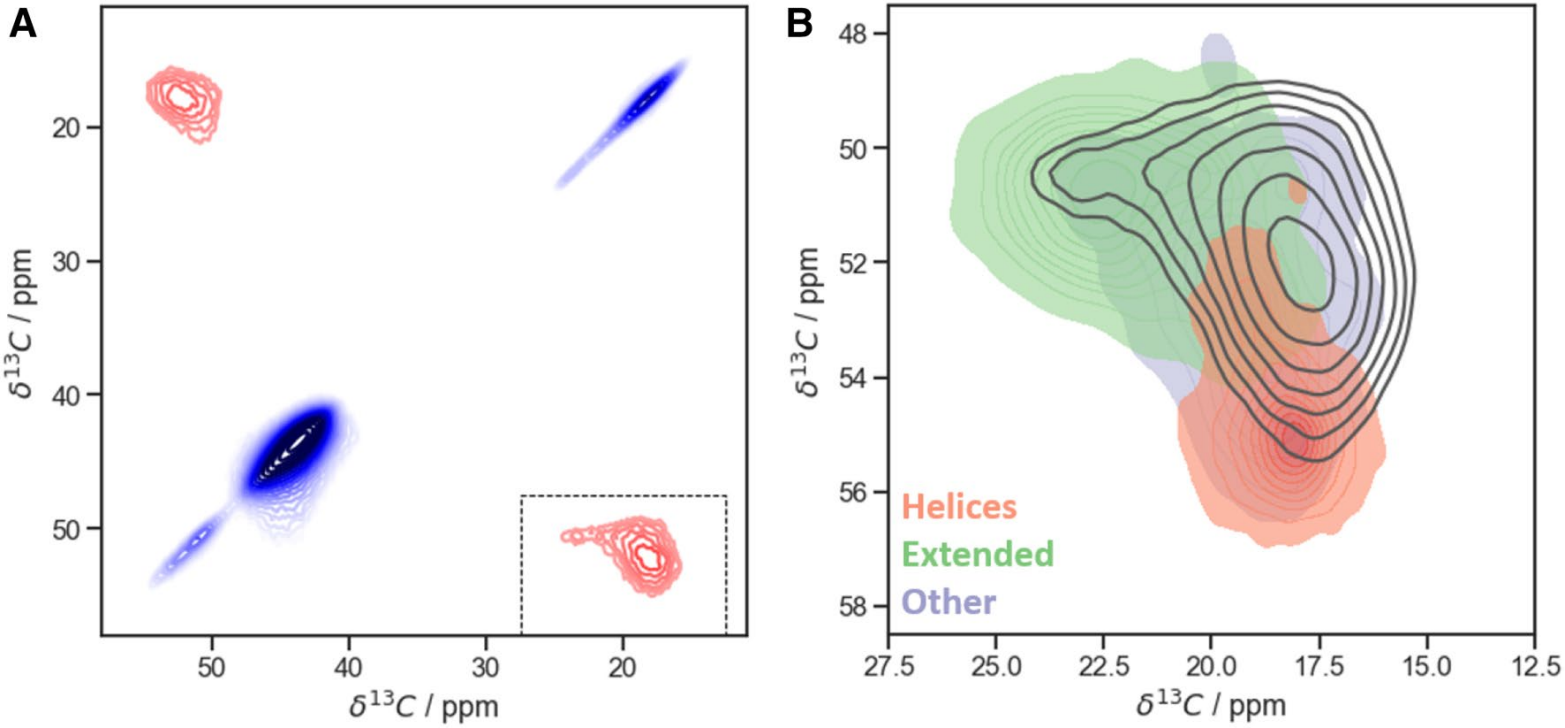
^13^C–^13^C 2D DREAM correlation of an inhomogeneous sample of GGAGG pentapeptide after freeze drying. **A** Full spectrum (line broadening coefficient LB = 20 Hz). **B** Overlay of ^13^C^α^/^13^C^β^ Ala cross-peak (black contours, with exponential line broadening of 150 Hz) with expected chemical-shift regions adopted by different kinds of secondary structure. These entries are color-coded by their secondary-structure class according to the STRIDE classification (Frishman and Argos 1995) with simplification: class “helices” includes alpha-, 3–10 and Pi-helices (H, G and I); “extended” class includes entries classified as E; other structures include the remaining T, B and b classes. Contours start from 4% of absolute intensity and increase with a factor of 1.2. Compare Fig. S4 for generation of secondary-structural color shades. Random-coil chemical shifts result from fast averaging of different conformations in solution and have been omitted here

As opposed to the previous works, we intended to utilize correlated chemical-shift data from as many nuclei as possible, expanding cross-polarization-based NMR experiments to four dimensions, which was put into practice via a 4D hCBCANH correlation. (See *Materials and Methods* and Table S1 for experimental details.) When applied to more complex proteins in the focus of biological questions in future studies, the 4D experiments will increase dispersion of the signals without individual site-specific labeling. Dispersion is a main bottleneck for the residue-resolved assessment of sample heterogeneity in case of severe peak broadening when many residues bear isotope labels. As expected, whereas the anti-correlated chemical-shift distributions of C^α^ and C^β^ (see carbon–carbon correlation in Fig. 2B) resemble what would be expected for a distribution of different dihedral angles based on the statistical data (Lee et al. 2012), the H and N distributions can be assumed to be more strongly influenced by homogeneous line broadening and heterogeneous contributions independent of dihedral angles. Therefore, in this preparation, these dimensions predominantly represent the purpose of chemical-shift dispersion. In DNP assessment of flash-frozen preparations (where intermolecular protein contacts are avoided due to an excess of solvent), however, H and N shifts may serve as a more faithful reporter on secondary structure as well.

### Conformational analysis based on predictions of TALOS-N

TALOS-N (Shen and Bax 2013) is a recent and widely-used program for predicting protein backbone dihedral angles from successive NMR chemical shifts that relies on an artificial neural network. The neural network ((φ, ψ)-ANN) is derived from proteins for which crystal structures are available together with their nearly complete backbone chemical shifts. The task of identifying the most likely (φ, ψ) combination for a given set of isotropic chemical shifts is closely related to the analysis desired here, where individual elements of the heterogeneously broadened peak need to be analyzed individually and an integrated distribution of angles be produced. In TALOS, the initial prediction of (φ, ψ) angle probability distribution is done with (φ, ψ)-ANN based on the chemical shift input for five consecutive residues. Then, the algorithm selects those 25 heptamer fragments with the best matching geometry and chemical shifts of the central residue to classify the result by quality and, if possible, find the most likely combination of (φ, ψ) angles.

**Fig. 3:**
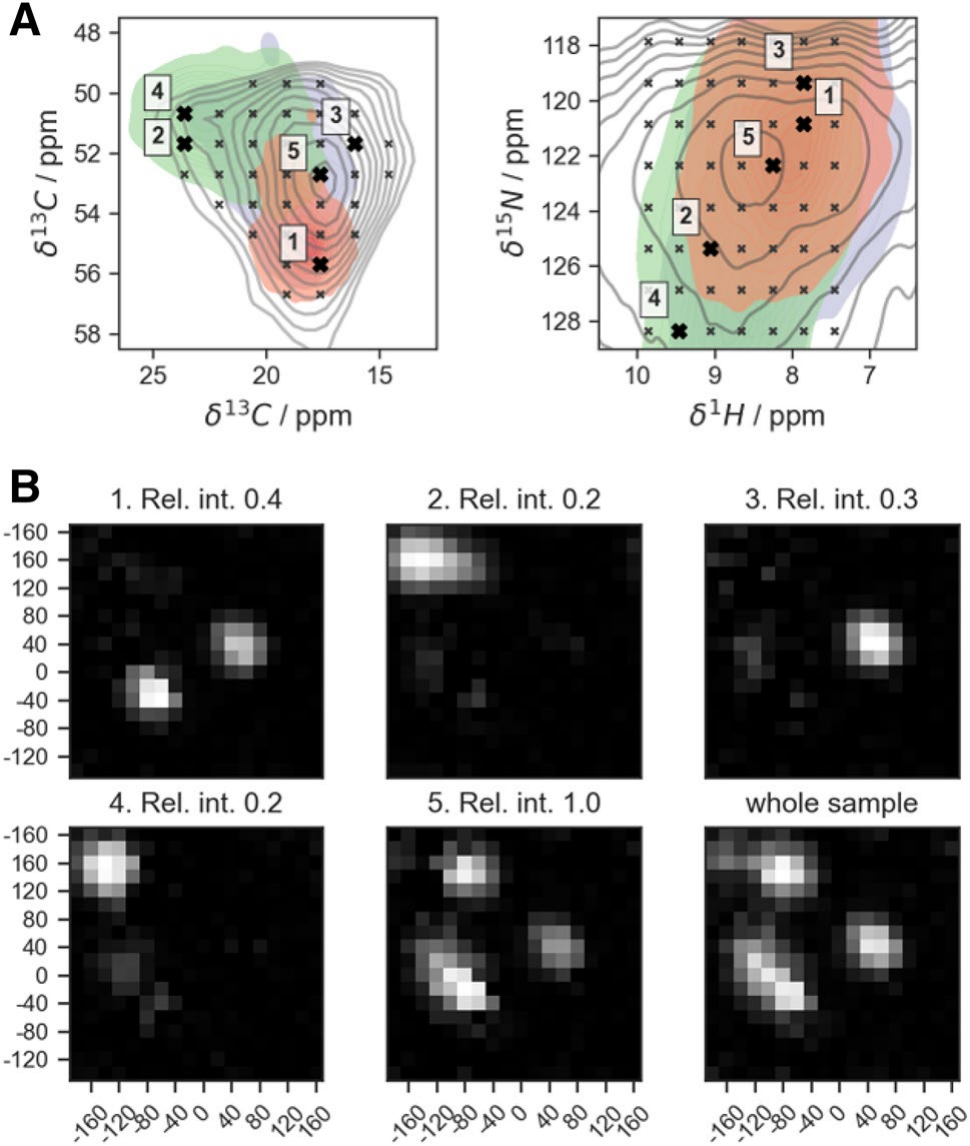
Process and results of TALOS analysis of the Ala cross peak in an hCBCANH spectrum of freeze-dried GGAGG. **A** Grid points within the peak (black crosses) and the selection of five test cases (“Points 1–5”, bold black crosses) over the orthogonal projections of the 4D peak (Cα/Cβ and H/N projection left and right, respectively) colored according to the chemical shifts expected for alanines (as occupied by 96% of the database entries, belonging to helical (red), extended structures (green), or other groups (purple)). Random coil (“C” class) entries have been left out—see caption to Fig. 2 and compare to Fig. S4. **B** TALOS predictions of the grid points defined in A (Points 1–5) for the extendend sequence, (G)GGAGG(G) (see main text), as well as the “whole sample”. The latter is the summed-up φ/ψ distribution map of all the grid points of the peak shape, representing the overall conformational ensemble of alanine in the inhomogeneous (G)GGAGG(G) sample

Given the explicit expectation of non-standard secondary structural properties, our TALOS-N based approach to static conformational disorder (see Fig. 2, right) utilizes only the 18 × 18 grid (φ, ψ) distribution obtained from (φ, ψ)-ANN (324 φ/ψ combinations). No other output data (the most likely (φ, ψ) combination, secondary structure propensity, prediction of side-chain rotamers) is used. To analyze the entire, heterogeneously broadened peak, the volume occupied by signal intensity above a given threshold (here: 20% of peak maximum intensity, signal-to-noise ratio of 15) was probed for intensity at discrete grid points of the chemical-shift space obtained via regular sampling intervals. Sampling resolution needs to be sufficient to differentiate the areas expected for different secondary structural properties (compare Fig. 3A). For the alanine cross-peak in GGAGG we used a spacing of 0.4, 1.0, 1.5, and 1.5 ppm in the ^1^H^N^, ^13^C^α^, ^13^C^β^ and ^15^N dimensions, respectively, corresponding to 1407 (4 + 1)D grid points (four frequency dimensions plus one dimension for intensity). The expected homogeneous linewidths (at 700–750 MHz and 40 kHz MAS) are on the order of 360, 20, and 80 Hz (0.5, 0.3, 0.5 ppm) in ^1^H, ^15^N, and ^13^C dimensions, respectively (Zhou et al. 2007; Linser et al. 2011). The inhomogeneously broadened lines of this sample (Fig. 3A), on the other hand, cover a shift range of several (~ 7–8) ppm in each dimension, which is in line with the expected large difference between helical and extended conformations present. As such, both the homogeneous contributions to the linewidth as well as the spacing of grid points are here sufficiently narrow in comparison to the extent of heterogeneous contributions but may have to be tightened in samples/residues with lesser extent of conformational heterogeneity.

In TALOS-N, secondary-structural assessment is strongly improved by including additional residues before and after the residue of interest (ROI). However, in the analysis of heterogeneous peak shapes, it is close to impossible to decipher which individual peak sections of the neighbor peaks are connected with which peak elements of the ROI, in the sense that they stem from the same molecule (or at least similar conformations). (Additional inter-residual dimensions, in addition to the multiple intra-residual ones, as well as added magnetization transfer steps would be required, which is practically challenging.) Simplifying the chemical shifts of neighbor spins to one value (e. g., the global peak maximum position), on the other hand, would strongly bias the analysis of the ROI to the most populated conformation. Therefore, we introduced a step of translating the ROI propensity associated with each given shift combination (grid point) to neighbor residue shifts (see Fig. 2) before subjecting the shift combination to ANN. For this purpose, the five-dimensional vector **r**_**i**_ between each grid point *i* and the neighbor-corrected random-coil chemical shift was determined for the ROI, appropriately rescaled, and added to the neighbor’s (*j* ± 1 and *j* ± 2) neighbor-corrected random-coil chemical shift values (Tamiola et al. 2010). Rescaling of **r**_**i**_ was done in nucleus- and residue-type-specific fashion that reflects the differential strength of shift modulation by secondary structure (Fig. S2), compared to the random-coil shifts, as determined using the conformation-specific centers of gravity for each nucleus and residue type (Fritzsching et al. 2016). For test purposes, we also artificially extended and translated the peptide chain by two glycine residues *j* ± 3 at the termini before prediction, as the TALOS-N database search involves up to 7-mers to make a prediction. Fig. S3A and B show a comparison of TALOS-N predictions for the true (GGAGG) and extended sequence (GGGAGGG) for the same test coordinates. However, the differences were found to be negligible. The analysis in the following was anyways done on predictions for the extended sequence. Assessment of 1407 points by TALOS took about 5 h (running 3 subsets of points in parallel on 10 Intel® Core™ i7-8700 CPUs at 3.20 GHz, 64 bit).

The grid of individual samples from the heterogeneous 4D peak is shown (as a 2D projection) in Fig. 3A, overlaid there with the regions expected for different types of different secondary structure. (See Fig. S4 for the generation of such secondary-structural regions.) The prediction results for five exemplary grid points selected to represent different contributions to the volume of the overall heterogeneous peak, i.e., two samples from the helical region (Points 1 and 3), two samples from the strand-like region (Points 2 and 4), and the point of the maximum overall peak intensity (Point 5), are shown in Fig. 3B, panels 1–5. Final reconstruction of the conformational distribution represented by the *overall* heterogeneous peak ${D}_{k}$ (the probability density at each of the 324 φ, ψ angle combinations *k*) is achieved by summing up the 1407 individual probability maps ${D}_{ki}$, weighted by the experimental intensity at each grid point ${I}_{i}$ in the experimental 4D spectrum (Fig. 3B, last panel, “whole sample”):

1 $$D_{k} = \sum\limits_{{i = 1}}^{N} {D_{{ki}} I_{i} }$$

with ${D}_{k}$—probability density for each φ/ψ combination $k$ on the Ramachandran map; ${I}_{i}$ is the NMR intensity at the position $i$ from the 4D peak volume (“grid point”), and $N$ is the number of grid points covered by the peak (in this case, $N=1407$). Note that we will use the variable ${D}_{k}$ for the height (probability) at a given point (*k*) in Ramachandran space irrespective of what the respective map looks like in detail.

**Fig. 4:**
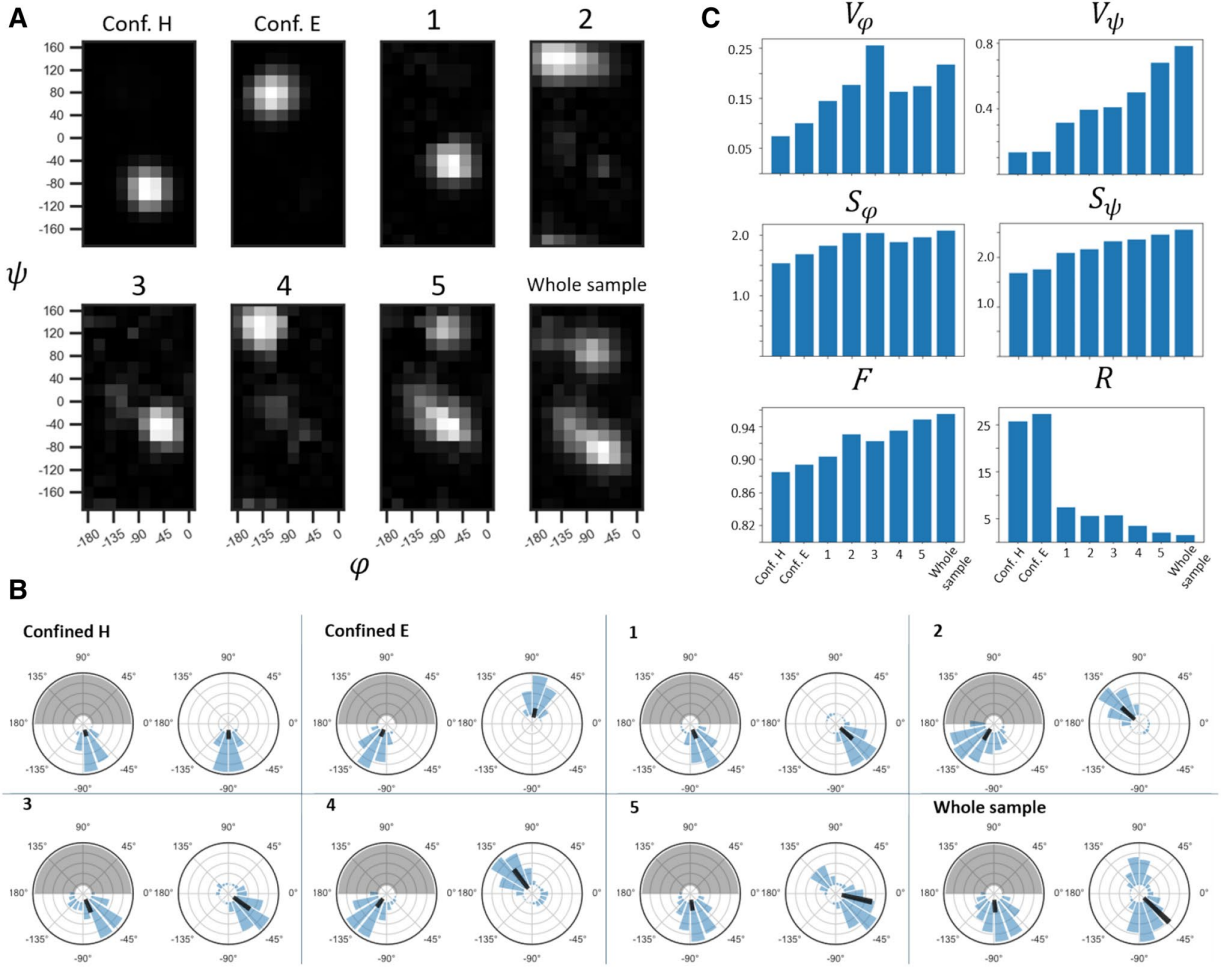
Exploration of various methods of quantification of heterogeneity in solid-state NMR samples, applied to TALOS-based reconstruction of conformational distributions. **A** Folded Ramachandran maps of the test coordinates. Panel “Whole sample” corresponds to the weighted sum of predictions over the whole Ala peak of heterogeneous GGAGG. For generation of pure secondary structure, predictions were made of the 5th Leu in a Leu_10_ chain with the corresponding expected chemical-shifts values (taken from (Fritzsching et al. 2016)). Grayscale is normalized from 0 (black) to 1 (white, maximum value). **B** Ramachandran maps from A) in polar coordinates. In each pair, the left plot corresponds to φ and the right one to ψ distributions. Gray area denotes the non-valid φ region for the calculations due to folding (see main text for details). Black vectors point into the *mean direction*, their length is set here to represent the circular variance, not the length of the resulting vector for the distribution. **C** Representation of different measures of heterogeneity (circular variance *V*, entropy *S*, flatness *F*, and secondary-structure ratio *R*) for the maps shown in A) as bar plots. See text for details

As expected from the shift distribution of GGAGG in comparison with neighbor-corrected chemical shifts resulting for different secondary structures (Fig. 2B), for this extreme case of static disorder, the individual predictions sample almost the whole allowed Ramachandran space (Fig. 3). Points 2 and 4 correctly represent extended conformation with *φ* = − 155 and *ψ* = 140, with some uncertainty for Point 2. Due to low relative intensity, these two points make only a minor contribution to the final result. Point 1, situated in the helical region of chemical shift distribution, correctly yields clear helical predictions. Interestingly, however, a mix of left- and right-handed helices is obtained. For Point 3, which is located in one of the turn regions, a left-handed helix is confidently predicted (or, according to classification in Hutchinson and Thornton (1994), a type I’ turn). In the case of an alanine surrounded by glycines—residues without any C^β^—both senses of winding are indeed possible and—given the enantiomeric character of the left-handed GGAGG helix relative to the right-handed one—would lead to similar chemical shift. Notably, overall, this sample contains more left-handed helical propensity than the conventional α-helical, according to TALOS-N (see panel “whole sample”). However, this is a special exception for this sample. (Accordingly, the PACSY database does not differentiate well between right- and left-handed helix, see below). Fig. S3 compares predictions for GGAGG with predictions at the same three test coordinates but for LLALL and LLLALLL as input and adjusting the shift translation by taking the Leu (instead of Gly) random coil values into account. In principle, this should give identical results since chemical shifts are equivalently translated, with the difference that the result is now in a purely *right-handed* helical conformation. This is indeed the case. Hence, in further analysis and for the general case, we omitted the sense of winding and considered *helical* contributions in a general sense. As such, the two different senses of helical properties derived from the shift combination at Points 1 and 3 were merged into single *helical* predictions by reflecting the right half of the resulting Ramachandran map onto the left one (point reflection about the 0, 0 coordinate, referred to as “folding” in the following, Fig. 4A).

Eventually, Point 5 yields a broader distribution of angles. It is associated with “dynamic” properties by TALOS-N and denotes overlapping contributions from turn (*φ* = − 80, *ψ* = 150) and any of the helical conformations (the region around (*φ* = − 90, *ψ* = − 40) for alpha- and 3–10 helices and (*φ* = 80, *ψ* = 10) for the left-handed helix). For completely rigidified samples, averaging of chemical shifts to pure random-coil values does not occur. Fig. S5A shows the entries from the PACSY data base, with the coil entries removed, colored according to dihedral-angle combinations (rather than the STRIDE system). Most of the turn conformations, with dihedral-angle combinations diverging from helical or extended conformations, are located in the central region of the chemical-shift space, similar to the coil shifts in solution. Whereas, within the turn conformations, the most extreme helical and sheet shifts are not adopted and certain trends are still obvious, the association between shift and angle combinations is much less clear than between the major classes of secondary structure (compare Figs S5C, E, and F, as well as Figs. S6 and S7). Hence, even though the occurrence of “neither-E-nor-H” cases in the data base is much lower than the more faithfully predictable E and H shifts, the ambiguity for predictions of intermediate shift combinations constitutes a well-known shortcoming of shift-based secondary structure prediction. Equally importantly, every point in frequency space can be thought of as reflecting contributions from individual molecules with their specific backbone dihedral angles. However, intensity in central positions will always occur also as an artifact from overlap of individual peak “shoulders”, to an extent dependent on the level of homogeneous contributions to the linewidth and limited digital resolution. Hence, in addition to the turn residues resonating with exactly these shift combinations (Fig. S5C and F), shoulders from the more helical and extended conformations (Fig. S5B and D) strongly blend into the contributions from the “turn” dihedral angles in this area. In this respect, the inhomogeneous prediction results for central shifts are not entirely wrong, as indeed they agree with the presence of a mixture. Hence, whereas the “mixed” prediction outcome for central shift combinations may have distortive character (when in reality a narrow distribution around one or more of the turn torsion angle combinations would be correct), a reasonably representative outcome can be expected for mixtures mainly comprised of different populations of the more prototypical (helical/extended) conformations. (In these cases, if not absent, central chemical shifts are derived from homogeneous line broadening.)

### Quantification of heterogeneity

Since there is no standard for the quantitative level of site-specific sample heterogeneity yet, here we explored different approaches to possibly represent the degree of heterogeneity in a fast and at least qualitatively reliable fashion. For this purpose, we considered eight distributions that represent a variety of possible scenarios: In addition to the "Points 1–5″ and the overall inhomogeneous peak shape (“whole sample”) considered above, two additional points that represent the “purest” homogeneous cases (expected helical and strand chemical shifts) are included in the comparison for convenience. (TALOS predictions for these pure helical and extended-structure cases were generated for the 6th residue of a Leu 10-mer sequence with chemical shifts being set to the expected values of either helix or strand (Fritzsching et al. 2016).) The eight scenarios are ordered tentatively from pure to mixed conformational content; however, it is clear that different measures (see approaches of quantification below) would be sensitive to specific features of the distribution. It may be useful to stress that, as a notion, the *degree of heterogeneity* makes sense only for the integral or “summed up” Ramachandran maps addressing the *whole* volume of a solid-state NMR peak (panel “whole sample” in Fig. 3B and the corresponding panel in Fig. 4A) and shall not be applied to the predictions from individual grid points of a heterogeneous peak (which were described in the previous section). In this section, we use the individual Ramachandran maps for demonstration purposes only, as if they were obtained for separate, homogeneous peaks, since all calculations remain exactly the same.

In order to characterise (φ, ψ) distributions quantitatively, at first, methods of circular statistics were applied. Each pixel in the Ramachandran map represents the (weighted) incidence of a specific angle combination. When generating a circular average over an angular distribution, one imagines an averaging of vectors in 2D space with an individual direction (given by the angle) and length (its weight within the ensemble). The (φ, ψ) distribution of the heterogeneous peak comprises of 180 vectors ${\overrightarrow{v}}_{k}$ (one for each (φ, ψ) combination *k*) with direction given by the pixel’s angles $\varphi$ and $\psi$ and magnitude given by the pixel’s relative intensity $\frac{{D}_{k}}{\sum_{k=1}^{180}{D}_{k}}$. Knowing the magnitude of an individual vector ${\left|\overrightarrow{v}\right|}_{k}$ and its angle(s), it can be dissected into vector components $\overrightarrow{\left|v\right|}\mathrm{sin}\theta$ and $\overrightarrow{\left|v\right|}\mathrm{cos}\theta$ using trigonometric relations ($\theta$ being $\phi$ or $\psi$ angles). The average vector $\overrightarrow{v}$ and its magnitude, $\left|\overrightarrow{v}\right|$, can then be constructed via Pythagoras from the average vector components. In the case of only similar angles being populated in Ramachandran space, the average vector $\overrightarrow{v}$ is long (in the extreme case has a magnitude $\left|\overrightarrow{v}\right|$ of 1, as the sum of relative intensities is 1), whereas with a large variety of angles being populated, the vector sum or average has a short length (in the extreme case 0). The *circular variance V* (with higher values for broader distributions and vice versa) is reflected by 1–$\left|\overrightarrow{v}\right|$:

2 $$\begin{gathered} V_{\theta } = 1 - \left| {\vec{v}} \right|_{\theta } = 1 - \sqrt {\left( {~\overline{{\left| {\vec{v}} \right|_{k} \sin \theta _{k} }} ~} \right)^{2} + \left( {\overline{{~\left| {\vec{v}} \right|_{k} \cos \theta _{k} }} ~} \right)^{2} } \hfill \\ = 1 - \sqrt {\left( {\frac{{\mathop \sum \nolimits_{{k = 1}}^{{180}} sin\theta _{k} \cdot D_{k} }}{{\mathop \sum \nolimits_{{k = 1}}^{{180}} D_{k} }}} \right)^{2} + \left( {\frac{{\mathop \sum \nolimits_{{k = 1}}^{{180}} cos\theta _{k} \cdot D_{k} }}{{\mathop \sum \nolimits_{{k = 1}}^{{180}} D_{k} }}} \right)^{2} } \hfill \\ \end{gathered}$$

${V}_{\theta }$ thus ranges from zero to one, where lower values correspond to concise distributions. Note that in our approach, the distibutions have a period of only 180° (π) for the φ dimension due to the above folding of left-handed into right-winded structures (see section *Conformational analysis based on predictions of TALOS-N*). The Ramachandran plots (Fig. 4A) can be visualized using polar coordinates (Fig. 4B), in which the distribution of vectors (blue histograms) and the average property (black) are visualized. Note that the magnitude of the black arrow was chosen to reflect the variance (1–$\left|\overrightarrow{v}\right|$), not the resulting vector’s magnitude. The rising trend of greater “inhomogeneity” in the individual panels (conf. H, conf. E, Points 1–5), which is apparent from both ${V}_{\phi }$ and ${V}_{\psi }$, shows the uncertainty within the prediction of individual chemical shift combinations: In contrast to the clear (easy-to-predict) shift combinations, an increasingly large *V* is found for Points 3–5, i. e., when shifts do not adhere to the standard values expected for helical or extended structures. This is consistent with the above observation that shifts in central regions are inherently associated with a broader (φ, ψ) distribution on their own.

Alternatively, the level of heterogeneity contained in broad (φ, ψ) angle distributions can be measured by *Shannon‘s entropy*. In statistics and information theory, the concept of entropy is widely used to quantify the amount of uncertainty in a given distribution of a random variable. Considering each φ/ψ combination $k$ of the Ramachandran map as an independent state of an amino acid residue, with its intensity *D*_*k*_ representing its likelihood to be true/adopted, the entropy of a prediction would be calculated as follows:

3.1 $$S_{\phi } = - \sum\limits_{{k = 1}}^{{10}} {D_{k} ln\left( {D_{k} } \right)}$$

3.2 $$S_{\psi } = - \sum\limits_{{k = 1}}^{{18}} {D_{k} ln\left( {D_{k} } \right)}$$

3.3 $$S_{{total}} = - \sum\limits_{{k = 1}}^{{180}} {D_{k} ln\left( {D_{k} } \right)}$$

Entropy of a hypothetical case where only one state is populated equals zero; by contrast, it increases up to $S=\mathrm{ln}\left(180\right)\approx 5.19$ for the hypothetical case of a uniform distribution.* If the angle-specific contributions to the entropy are of interest, they can be calculated via projection of the Ramachandran probability map onto the individual axes and applying the above routine (then $0\le \mathrm{k}\le 10$ for $\varphi ; {S}_{\varphi }^{max}=\mathrm{ln}\left(10\right)\approx 2.3$ and $0\le \mathrm{k}\le 18$ for $\psi ;$ ${S}_{\psi }^{max}=\mathrm{ln}\left(18\right)\approx 2.9$). When describing the full heterogeneous peak, the Ramachandran map ${D}_{k}$ refers to the result from weigthed averaging over the heterogeneous chemical-shift pattern (see Eq. 1). For the heterogeneous GGAGG sample of this study, the total entropy *S*_total_ is 4.46, whereas entropy values of individual (one-dimensional) $\varphi$ and $\psi$ distributions amount to 2.07 and 2.56, respectively. Note that in Eq. 3.3, *D*_*k*_ (the probability for the φ/ψ *combination k* in the Ramachandran map) applies to the *folded* map with *k* = {1, …, 180}. For single-angle entropies (Eq. 3.1 and 3.2, *k* bearing 10 or 18 values for *φ* and *ψ*, respectively), *D*_*k*_ refers to probabilities for *individual* *φ* or *ψ* values in one-dimensional Ramachandran maps. (Such projections are obtained by adding all those *D*_*k*_ values that are within the same column or row, respectively). It may be useful to correct for the level of ambiguity of the prediction for a well-defined event, which *excess entropy* results from simple subtraction of the entropy for a confined helix: $\Delta S=S- {S}^{conf. H}$. For the heterogeneous GGAGG sample, the overall $\Delta S$ amounts to 1.30, the highest-possible (but sterically challenging) value would be 2.02.

A simple approach to probe the level of homogeneity found in a distribution is the measure of *flatness*, which gives the relative abundance of the highest-probability event (normalized by the sum of overall occurrence of different events of the prediction):

4 $$F=\frac{\mathrm{max}\left({D}_{k}\right)}{\sum_{k=0}^{180}\left({D}_{k}\right)}$$

**Fig. 5:**
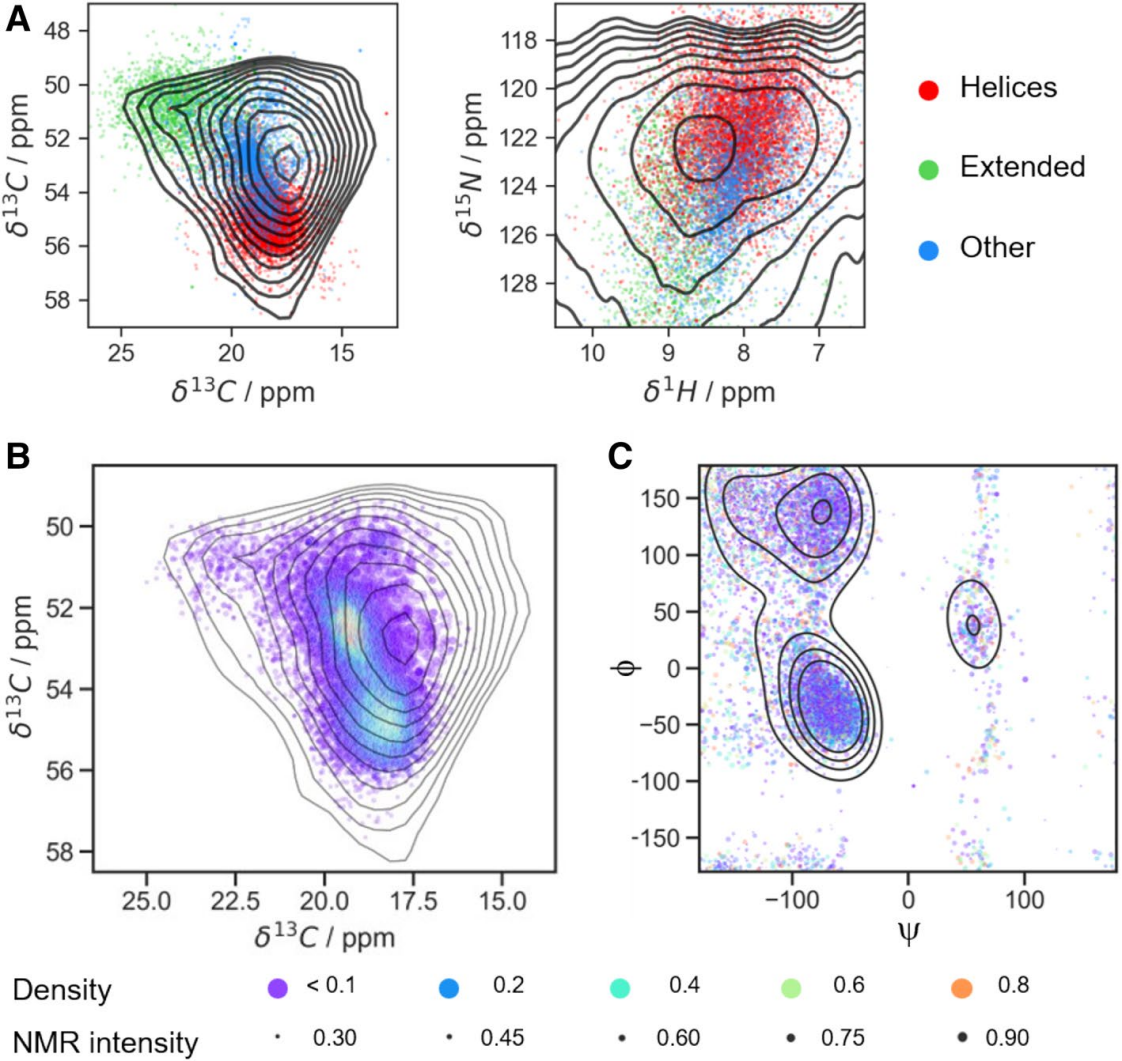
Selection of points and results of PACSY-based assessment of conformational heterogeneity in the GGAGG sample. **A** Representations of entries from the cleansed PACSY database for Ala, color-coded by their secondary-structure class according to the STRIDE classification (Frishman and Argos 1995) with simplification: class “helices” includes alpha-, 3–10 and Pi-helices (H, G and I); the “extended” class includes entries classified as E; other structures include the remaining T, B and b classes; random coil entries were excluded from the presentation. Left: ^13^C^α^ / ^13^C^β^ and right: ^1^H/^15^N projections of the data base and 4D hCBCANH spectrum. Contours start from 4% of absolute intensity and increase with a factor of 1.2. **B** Points from the data bank that belong to the 4D volume of the Ala cross-peak in the hCBCANH spectrum; Gray contours depict a bivariate weighted kernel density estimate: The weight of each point is a product of interpolated intensity of the 4D peak and the inverse point density in the PACSY database in the 4D chemical shift space. (Colors, as denoted in the bottom, refer to original (non-inverted) data bank entry density.) Contours start at 15% density and succeed with a factor of 1.1. **C** The same entries plotted in φ/ψ space

By definition, it is insensitive to the number of modes and rather characterizes how confined the distribution is overall (Fig. 4C). In addition, it may be interesting to consider the *ratio between the population of helical and extended regions* (*R*), as determined from the integral over relative densities in the typical areas of the Ramachandran plot.

5 $$R=\left\{\begin{array}{cc}H/E& if\,\, H>E;\\ E/H& if \,\,H\le E\end{array}\right.$$

where H and E are the integrals of the allowed regions in the folded φ / ψ maps. (Regions taken into account for H and E are depicted in Fig. S8.) Tables 1 and 2 and Figs. 4 and 5 show *R* for various cases. For the whole sample, *R* amounts to ~ 1.53, with a slight excess of helical properties.

**Table 1:** Heterogeneity parameters obtained for the folded Ramachandran maps predicted by TALOS-N for the local test scenarios as well as for the broad heterogeneous peak. Shown are the two reference cases (H and E), five individual coordinates from the Ala HNCACB peak (“Points 1–5"), and the cumulative peak volume in the GGAGG sample (bold, see Fig. 4A). The underlining in the sec. structure column denotes the excess of helical content

Scenario	Sec. struct	Circular variance *V*		Entropy *S*				Flatness *F*	*R*
		*ϕ*	*ψ*	*ϕ*	*ψ*	total	Δ*S*_total_		
Conf. H	H	0.07	0.13	1.54	1.68	3.17	0.00	0.886	25.89
Conf. E	E	0.1	0.14	1.68	1.76	3.35	0.18	0.894	27.38
Point 1	H	0.15	0.32	1.82	2.10	3.68	0.51	0.904	7.53
Point 2	E	0.18	0.40	2.04	2.17	4.08	0.91	0.943	7.31
Point 3	H	0.26	0.41	2.04	2.33	3.99	0.82	0.923	5.80
Point 4	E	0.16	0.5	1.88	2.37	3.99	0.82	0.935	3.55
Point 5	H + E	0.18	0.68	1.96	2.47	4.24	1.07	0.949	1.99
**Whole sample**	**H + E**	**0.22**	**0.78**	**2.07**	**2.56**	**4.46**	**1.30**	**0.955**	**1.53**

**Table 2:** Quantitative analysis of Ramachandran maps obtained using the PACSY approach, focusing on chemical-shift combinations of confined helix and sheet, Points 1–5, and the full heterogeneous GGAGG peak (bold). Since for the PACSY approach in clean cases no population of incorrect secondary structure is produced, the *R* values tend to be infinity (division by 0) or very high, which hence represents a clean prediction. *N* stands for the number of PACS entries at the respective chemical-shift grid point. The underlining in the sec. structure column denotes the excess of helical content

Scenario	Sec. struct	N	Circular variance V		Entropy *S*				Flatness *F*	*R*
			***ϕ***	***ψ***	***ϕ***	***ψ***	Total	Δ*S*_total_		
Conf. H	H	2030	0.04	0.06	0.96	1.16	2.02	0.00	0.666	115.9
Conf. E	E	105	0.12	0.08	1.70	1.55	3.04	1.02	0.911	inf
Point 1	H	1303	0.02	0.04	0.88	0.99	1.83	− 0.19	0.645	419.6
Point 2	E	60	0.09	0.09	1.51	1.45	2.61	0.59	0.786	inf
Point 3	H	6	0.28	0.22	1.36	1.09	1.36	− 0.66	0.671	0.72
Point 4	E	20	0.06	0.07	1.31	1.0.37	2.26	0.24	0.790	inf
Point 5	H + E	422	0.17	0.74	1.64	2.45	3.71	1.70	0.860	1.54
**Whole sample**	**H + E**	**13,565**	**0.17**	**0.80**	**1.78**	**2.36**	**3.94**	**1.92**	**0.869**	**1.52**

**Fig. 6:**
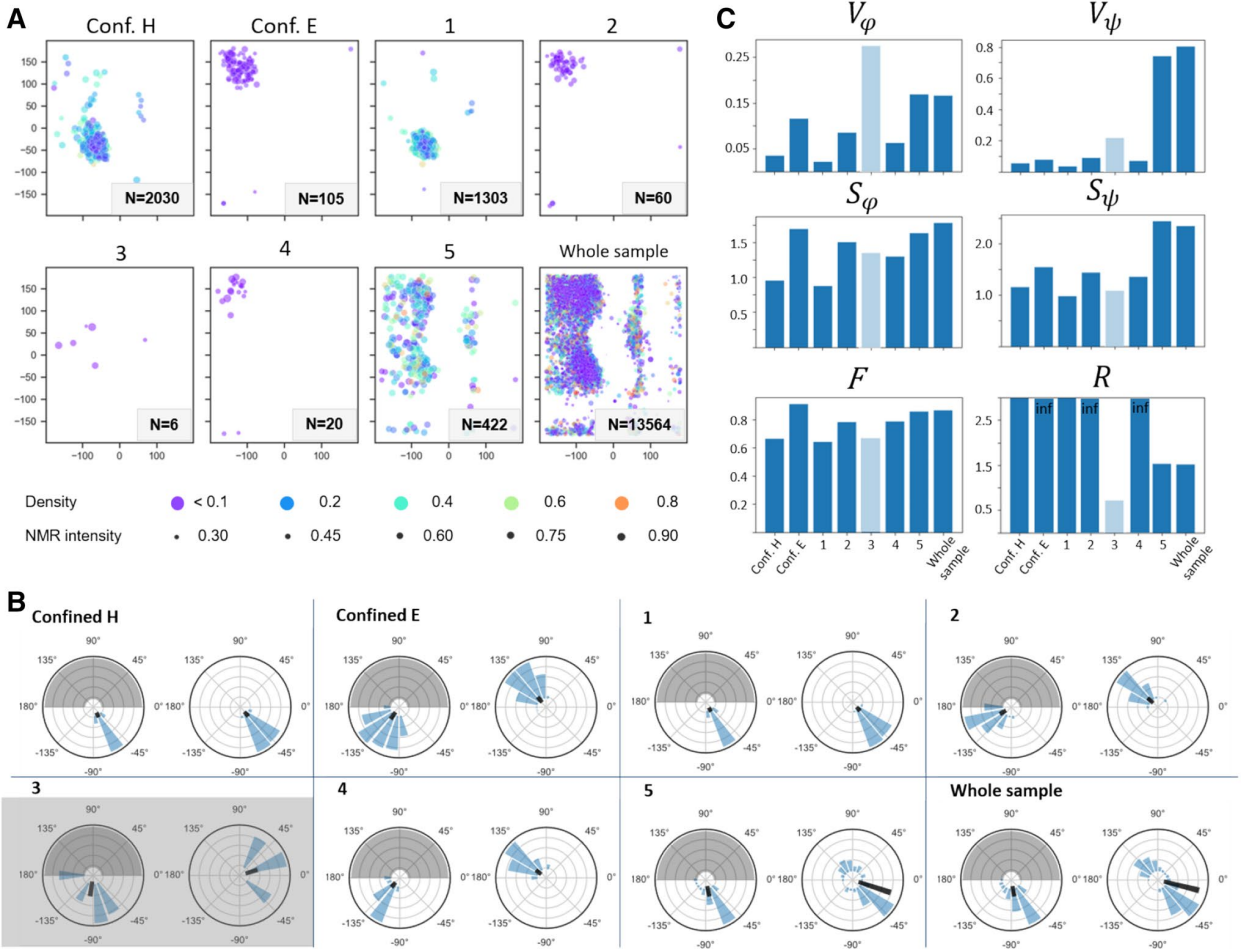
Distributions of dihedral angles for the entries of PACSY database selected by their chemical shifts to represent the scenarios considered in TALOS-based approach and their parameters. **A** Sets of entries in φ/ψ space. Width of each interval is 0.8 ppm for ^1^H, 2 ppm for ^13^C^α^, and 3 ppm for ^13^C^β^ and ^15^ N dimensions. Selection for the entire heterogeneous peak as described in the main text. **B** Same distributions of PACSY entries, folded and represented in polar coordinates. In each pair, the left and right plot represent the individual φ and ψ distribution, respectively. Black bars represent circular variance and point to the mean direction. Entries around Point 3 are grayed out because they comprise too few points (N = 6). **C** Heterogeneity parameters for each set: circular variance *V*, entropy *S*, flatness *F*, and secondary-structure ratio *R* (see main text for the formulae). Results for the set around Point 3 are again bleached out due to too few database entries

### Conformational analysis based on predictions of DANGLE

As an alternative approach to neural-network based tranformation of chemical-shift combinations into dihedral angle space, the DANGLE algorithm has been suggested (Cheung et al. 2010). DANGLE uses Bayesian inference-based methodology and was set up in 2009 for improving predictions over the TALOS-N predecessor TALOS + (Shen et al. 2009) at the time. We subjected the 1407 chemical-shift combinations covering the heterogeneous 4D peak derived above also to DANGLE, using exactly the same procedure as described in the framework of TALOS predictions. Quantitative evaluation of the predictions of the exemplary grid points described above (confined H, confined E, and Points 1–5) as well as the summed prediction for the entire heterogeneous peak was also done as described above (Fig. S9 and Table S3). A series of DANGLE test runs where—as an alternative—the secondary-structure propensity of the middle residue (Ala) was *not* propagated to its neighbors (all four Gly) yielded identical φ/ψ maps for all grid Points 1–5. With translation of secondary-structural propensity to neighbors (determining the deviation between the grid point shift combination to the random-coil shift combination and calculation of neighbor shift combinations from their random-coil shifts by adding the same difference as found for the ROI, as described above), however, results were obtained that are very similar to the TALOS approach. In particular, the expected angular properties are faithfully reproduced for confined H, confined E, and Points 1, 2, 4, and 5. A deviation is found only for grid point 3, which represents a rather sparsely populated area in chemical-shift space and also fails to yield faithful predictions in the approach based on direct data base correlations (see below). Generally, however, the individual predictions seem more discrete, and lower variability in the prediction results from individual chemical-shift grid points is found. As a consequence, whereas the trends within the data set are qualitatively consistent with the expectation and with the TALOS-N approach, the exact values found in the various heterogeneity scores of the overall peak are generally lower. In contrast to the prediction by TALOS, where statistics are generally smoother, many zero values are obtained, and added care has to be taken to not overinterpret the resulting quantitative scores.

### Conformational analysis driven by database search

A rather different approach to reconstructing the ensemble of conformations from a heterogeneously broadened peak shape is the utilization of a database that directly associates backbone dihedral angles from PDB structures with chemical shifts. We used PACSY (Lee et al. 2012), a relational database that contains over 6000 protein chains to allow this correlation directly from individual entries. For this purpose, we constructed the following workflow (see Fig. 2, left). All data are taken from the PACSY table X_CS_DB2 (where X stands for one-letter residue code, here X = A), which relates chemical shift, dihedral angles, secondary structure classification (according to STRIDE algorithm (Frishman and Argos 1995)), and other information. Residues that belong to the proteins marked as not passed PIQC were excluded from further analysis. All remaining entries within the populated 4D area of chemical shifts (the peak envelope) were used to reconstruct the underlying conformational ensemble. In order to compensate for the random, shift-combination-specific sparsity of data bank entries and avoid the resulting bias that varying entry densities would have for reconstructing the ensemble, the elements were weighted in height by the inverted local 4D entry density. Then, to also reflect the actual peak shape (the experimental distribution of shift contributions), final weighting of the *i*th entry amounts to ${w}_{i}={I}_{i}\bullet {P}_{i}^{-1}$, where ${I}_{i}$ is the peak intensity and ${P}_{i}$ the density of data bank entries at the respective chemical-shift positions. The selected points are shown in Fig. 5 with colors of the points corresponding to the original entry density and sizes corresponding to the relative intensity of the 4D peak; Fig. S10 shows the density map without normalization by point density for comparison. The reconstructed conformational ensemble is represented by the weighted entries depicted in the φ/ψ map (Fig. 5C). As an experiment, we calculated all mathematical scores for the extent of heterogeneity presented in the section *Quantification of heterogeneity* also for the PACSY approach. In order to represent test Points 1–5 (compare respective panels in Fig. 4A) we selected entries belonging to an interval centered at each of these chemical shift combinations (Fig. 6A). The interval was chosen equal to twice the TALOS grid resolution to represent a larger number of data bank entries (i. e., 0.8 ppm in ^1^H, 2 ppm in ^13^C^α^, and 3 ppm in both, ^13^C^β^ and ^15^ N). Test Point 3 (Fig. 3A) appeared so far away from the main clusters of PACSY entries that it included only 6 points and was excluded from further consideration; all the results are, however, still presented here for consistency (Fig. 6). “Clean” cases of confined helix and extended structure were represented here with a selection belonging to the box centered around the mode for chemical shifts of, correspondingly, H- and E-classified alanines. Finally, the selection for the entire heterogeneous peak (“whole sample”) was made as described above.

Heterogeneity scores were calculated for the weighted and folded 10 × 18 maps converted to the format of the TALOS-based approach (Fig. S11). As in Fig. 4C, the bar charts in Fig. 6C (center row) only display absolute entropies, whereas in Table 2 we also list excess entropy for the entire Ramachandran map, which is the difference of individual scenarios from the case of a clean helical shift taken again as a reference.

Compared to the TALOS-based procedure, the obtained results for the heterogenized GGAGG sample are in fact similar. Each of the individual grid points yields a prediction qualitatively consistent between the methods as well as in line with what is expected from the known chemical-shift trends depicted in Fig. 3A. For the entire peak, the expected broad conformational distribution with the highest contribution for the angles corresponding to extended and helical conformation as well as a slight excess of helical conformations applies again. Even the quantitative assessment of heterogeneity across the full peak is highly consistent, which is gratifying to see given the various shortcomings for purely shift-based reconstruction mentioned throughout the manuscript. E.g., the parameter *R*, the ratio of populations associated with helical conformations and those associated with strand-like conformations, for the entire peak is 1.52, which is identical to the value obtained for TALOS-based maps (Table 2). The distribution of *ϕ* and *ψ* values adopted as measured by circular variance compares as 0.17 versus 0.22 and 0.80 versus 0.78, respectively. The excess entropy compares as 2.07 versus 1.78 and 2.56 versus 2.36 for *ϕ* and *ψ*, respectively. (Theoretically possible values for the 10 × 18 histogram lie between 0 and 2.3 for *ϕ* and between 0 and 2.9 for *ψ*.) Applying the scores for determining the “degree of heterogeneity” to the individual chemical-shift grid points—which is physically insensible—the trends are reasonably consistent and confirm the better predictability of more common vs. uncommon shifts. (Such test should in ideal cases yield low values and is expected to give more inhomogeneous predictions only for shift combinations outside the clean E or H conformations.) However, per pixel, only the predicted secondary structure is of interest in future applications, and the heterogeneity scores would be applied only to a whole heterogeneous peak. Eventually, future applications would always compare different residues of the same sample within the same methodology, and indeed these trends are fully in agreement with the expectation for all approaches tested.

## Discussion

Even though different approaches have been proposed in the past, a detailed description of a conformational ensemble within a single sample has remained difficult to obtain. In previous studies, many of which are listed in the introduction, experimental means could often be used to disentangle disorder into individual samples for a reconstruction of their conformational properties. Alternatively, only few discrete conformations co-existed. In both cases, tailored spectroscopic methodology could be used to shed light on their site-specific properties. Limitations to such approaches occur in the case when the biological sample cannot be physically disentangled. The same applies when broad distributions within the data are expected that are not resolved via the chemical-shift space available. Ensemble properties from such heavily overlapping patterns have been reconstructed in other approaches from accordingly, *a-posteriori* reweighted conformational ensembles, obtained from MD in conjunction with shift prediction. Although this represents a very elegant approach, even with perfect performance of the available chemical-shift prediction tools, such data patterns can be underdetermined, leading to multiple (different) ensembles being in reasonable agreement. This is even more likely in case of peak overlap, which causes high-complexity distributional properties of multiple sites to be entangled in a single, lower-complexity pattern.

Using chemical shifts as a direct reporter of dihedral-angle properties is a rather straightforward and hence sensitive approach that dispenses encoding of angular features by dedicated pulse sequence elements. This, conversely, facilitates the addition of multiple chemical-shift dimensions for peak dispersion and voxel-specific interrogation. Higher-dimensionality chemical-shift correlations in the framework of proton detection in particular bear the prospect of creating sufficient space for peak dispersion, along with a high signal-to-noise ratio from small sample volumes. This largely facilitates a residue-specific assessment of disorder in solid preparations as a function of sequence that would be overlapped using lower dimensionality. The above data show that the correlation between shifts and dihedral angles is reasonably trustworthy for the extreme cases of secondary structure for carbon nuclei, which facilitates a faithful reconstruction of the secondary-structural distributions within a heterogeneously broadened but non-overlapped peak. In congruency with the longstanding shortcomings of shift-based dihedral-angle prediction for homogeneous samples, however, the data also show that reliable reconstruction of heterogeneity around “intermediate” angle combinations (turn structures) is compromised by the general uncertainty of chemical shifts adopted in these cases—even when other factors modulating the shift are ignored. A good prediction will hence be obtained when the relative amount of helix and sheet is to be determined, whereas a poor prediction may arise when a residue comprises a narrow angular distribution around an intermediate angle combination that is difficult to interpret.

We expect that, often, the level of accuracy obtained for individual ensemble members within this framework is sufficient to answer biological questions related to heterogeneous conformational distributions. Examples would be to residue-specifically quantify the ratio of extended to helical conformers within amyloid preparations, flash-frozen folding intermediates, or rigidified disordered parts within membrane proteins. In such cases, the approach enables analysis with relatively low effort and costs. In addition, analysis of all residues within one preparation without selective labeling also bears the advantage of avoiding differences between samples (Xiang et al. 2017), which can compromise a consistent analysis. The maximal length of the primary sequence that can be subjected to the approach with reasonable dispersion and measurement time depends on the degree of heterogeneity. This derives from the fact that wider peak shapes both, increase the probability of overlap and the signal to noise of the resultant data. Luckily, in most of the current studies on samples of biological interest by NMR, only part of the residues tend to be variable, which reduces the probabily of overlap even for longer primary sequences.

The voxel-specific, higher-dimensionality methodology, in particular sample preparation/requirements and hardware involved, is substantially different from any of the carbon-detected approaches developed previously. As such, the results obtained here from the comparative implementation of prediction and direct data base approaches were only mutually validated by comparison with each other. Importantly, the introduced scores are designed as a measure for the *sequence-specific* degree of torsional variability over the ensemble, comparing different residues or samples within the same setting. Hence, self-consistency (i.e., the relative degree of disorder) within a single method is the most crucial property. A truly orthogonal (experimental) method would be desirable to benchmark or even just validate the findings from a non-NMR perspective. Unfortunately, however, methods that yield faithful distributions of conformations in mixtures are rare. In fact, various other biophysical techniques can principally be utilized to verify or support MD and NMR results. However, it is very difficult, if not impossible, to experimentally assess *site-specific* properties of *individual ensemble members* outside of NMR. Even when different conformations imprint themselves in the overall data, as for circular dichroism, powder diffraction, or FTIR, the obtained patterns are usually not sufficiently specific to reconstruct the underlying ensemble with residue resolution. The major alternatives to NMR as high-resolution structural-biology methods, cryo electron microscopy (cryoEM) and single-crystal X-ray diffraction, by contrast, may only deal with/quantify a limited degree of disorder (Nwanochie and Uversky 2019).

Despite the encouraging methodological results described above, in both approaches, however, the well-known systematic and general shortcoming of using chemical shifts for assessing torsional properties is the inherent sensitivity of the chemical shift to various physical effects other than backbone dihedral angles. A first factor is the influence of direct spin–spin interactions (homogeneous contributions) to the proton line. However, the role of protons will be largely restricted to further dispersing the peaks, given the rather loose association between their shifts and dihedral angles. The potential impact of differential contacts with the lattice represents a source of additional peak broadening potentially involving all nuclei. However, this drawback is expected again more strongly for those nuclei involved in H-bonds (H/N), whereas carbon shifts are more faithful reporters on angular properties. In particular, the *pair* of C^α^/C^β^ shifts, probed in chemical-shift-based approaches usually as a shift *combination*, bears opposite secondary chemical-shift trends and is mostly influenced by dihedral angles. In fact, the populated shift space in the carbon/carbon plane, which reflects the anti-correlated trends of C^α^ and C^β^ for secondary structure both for the heterogeneous peak of this study as well as the data base entries (Figs. 2 and 6A), speaks against large additional contributions to the carbon pattern, both for our test sample as well as in general. This renders the chemical-shift-correlation approaches here (be it via prediction or data base matches) more resilient to other influences compared to the inhomogeneous chemical shift of a single dimension. Differential sidechain torsional angles, however, can be an added source of shift modulation. This effect is expected for fully rigidified, longer side chains (opposed to only the protein backbone or Ala residues) and is difficult to disentangle from secondary-structural modulation of the shift (Siemons et al. 2019). Lastly, on purpose, aromatic moieties, chemical or magnetic perturbations (e. g. differential oxidation states in cysteines/pseudo contact shifts etc.) were avoided for this low-molecular-weight peptide, as these can have longer-range effects on the chemical shifts of close-by nuclei (and in this case molecules). The C^α^ /C^β^ shift pair as the most informative/convergent source of information both for the prediction as well as for the data base approach will mostly be influenced in a similar way by nearby ring currents, such that the shift difference remains largely untouched. Nevertheless, residues in close vicinity of aromatics probably need to be treated with care. In this special sample, additionally, only a low level of residual solvent is likely present, which renders the H-bonding properties of the amides rather variable. For future samples with a high content of solvent, on the contrary, more consistent hydration properties of polar groups and larger spatial separation of individual molecules are expected. As such, the approach could turn out particularly useful for site-specific insights in solidified systems with lots of frozen water (e.g., from flash-freezing without lyophilization), looked at by DNP. At least as a qualitative measure for the degree of how defined conformational properties are within a given primary sequence, the described methodology should turn out helpful and—given the availability of all Python-based workflows as a download—easy to set up.

## Conclusion

We have proposed a higher-dimensionality NMR approach to assess the site-specific conformational content in heterogeneous samples. Employing a single 4D hCBCANH spectrum, we enable heterogeneously broadened peak shapes in which the chemical shifts from individual conformers are correlated in the sense of a shift quadruple, from which the distribution of *ϕ*/*ψ* dihedral angles present for a given residue can be reconstructed. We demonstrate this reconstruction by two approaches, in a dihedral angle prediction-based and a data-base-derived manner, which—within the general limitations of shift-angle correlations—allow for reconstruction of the conformational distribution, in particular the ratio between sheet-like and helical conformers, of each residue that can be separated from other residues with four chemical-shift dimensions. As carbon shift combinations (i. e., the C^α^/C^β^ shift difference) are comparably weakly affected by contributions other than secondary structure, the approaches should represent both, a reasonable measure for qualitative but self-consistent assessment of conformational heterogeneity, as well as enable sufficient dispersion for assessing inhomogeneity of proteins as a function of sequence without specific labeling. This may facilitate probing site-specific conformational heterogeneity in whole amyloids and freeze-trapped samples from protein folding. The mathematical approaches to analyze *ϕ*/*ψ* distributions shown here may be useful for quantification of variability in conformational ensembles of future research in general.

## Supplementary Information

Below is the link to the electronic supplementary material.

Supplementary file1 (PDF 3098 kb)

## Data Availability

The NMR spectrum analyzed during the current study is available from the corresponding author on reasonable request. All scripts along with sample data will be made available from the journal web site for facilitated future application.
