# Supplementary material for: Characterization of conformational heterogeneity via higher-dimensionality, proton-detected solid-state NMR
Source: J Biomol NMR. 2022 Sep 23;76(5-6):197–212. doi: 10.1007/s10858-022-00405-0 (PMC9712413; doi:10.1007/s10858-022-00405-0)
Supplement: Supplementary file 1 — Supplementary file1 (PDF 3098 kb) [file 10858_2022_405_MOESM1_ESM.pdf]

Supporting information for

Characterization of conformational heterogeneity via higher-dimensionality, proton-detected solid-state NMR

by Ekaterina Burakova[1,2], Suresh K. Vasa[1,2], Rasmus Linser[1,2,*]

[1] Department of Chemistry and Chemical Biology, Technical University Dortmund, Otto-Hahn-Str. 4a, 44227 Dortmund, Germany

[2] Department of Chemistry and Pharmacy, Ludwig-Maximilians-University Munich, Butenandtstr. 5-13, 81377 Munich, Germany

*: Corresponding author, E-mail: rasmus.linser@tu-dortmund.de

Tables

Table S1: Acquisition and processing parameters of the 4D hcaCBCANH experiment

	^{13}Cb (F1)	*^{13}Ca (F2)*	*^{15}N (F3)*	*^{1}H (F4)*
Base frequency, MHz	201.24	201.24	81.1	800.3
Number of points	2x48	2x48	2x21	2048
Spectral width, ppm	59.9	59.9	33.1	26
Offset, ppm	14.1*	29.0	112.5	4.40
Apodization	Exp, 200	Exp, 200	Exp, 400	Exp, 500
Zero filling up to, points	128	128	128	2048
DREAM pulses	Tangential, 75%; 35.9 kHz		-	-
NUS points	2401 (ca. 5%)			
Number of scans	56			

*offset for the DREAM pulse and during evolution time

Table S2: Parameters of Ala crosspeak and its TALOS evaluation

*rectangular grid; before exclusion of non-intense and noise points

		**^{1}H**	**^{15}N**	**^{13}Ca**	**^{13}Cb**
Line width	Hz	2300	670	829	567
	ppm	2.8	8.2	4.12	2.82
Sampling grid resolution, ppm		0.4	1.5	0.8	1.5
Number of points*		7	11	9	9

Table S3: Parameters obtained in quantifying DANGLE predictions. The R score becomes infinity for many of the Points due to the devision by zero.

Scenario	Sec. struct.	Circular variance *V*		Entropy *S*				Flatness *F*	*R*
		ϕ	ψ	ϕ	ψ	total	ΔS_{total}		
Conf. H	H	0.01	0.00	0.52	0.11	0.62	0.00	0.24	inf.
Conf. E	E	0.00	0.00	0.04	0.19	0.23	-0.39	0.53	inf.
Point 1	H	0.01	0.01	0.31	0.61	0.88	0.26	0.39	inf.
Point 2	E	0.00	0.06	0.17	0.77	0.91	0.29	0.37	40.2
Point 3	H	0.01	0.02	0.55	0.80	1.23	0.61	0.54	inf
Point 4	E	0.09	0.03	1.50	0.99	2.39	1.77	0.79	inf
Point 5	H + E	0.00	0.01	0.05	0.51	0.55	-0.07	0.21	364.5
**Whole sample**	**H+ E**	**0.08**	**0.80**	**1.31**	**1.70**	**2.52**	**1.90**	**0.82**	**1.17**

Supplemental Figures

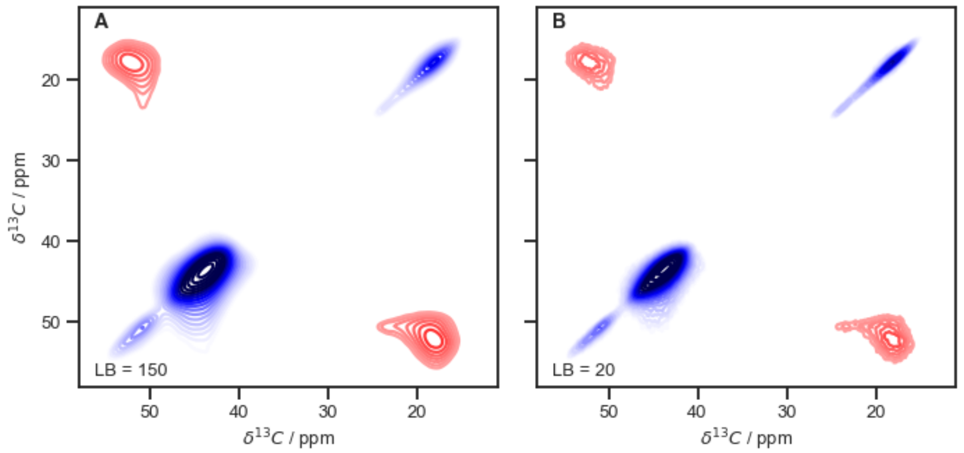

Fig. S1: 2D ^{13}C-^{13}C spectra processed with different line broadening coefficient (LB). A) LB = 150; B) LB = 20 (duplicated from the main text, Fig. 1a for clarity of comparison)

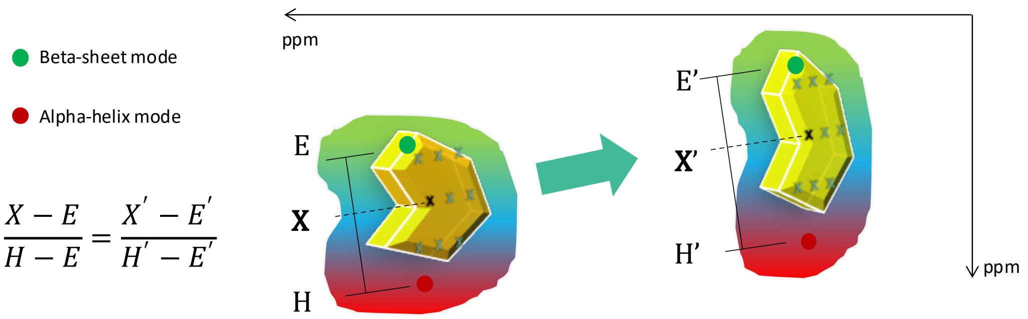

Fig. S2: Sketch of translation of peak of the Residue Of Interest into space, characteristic for neighbors' residue type. Polyhons depict multidimensional NMR peak for residues of different types

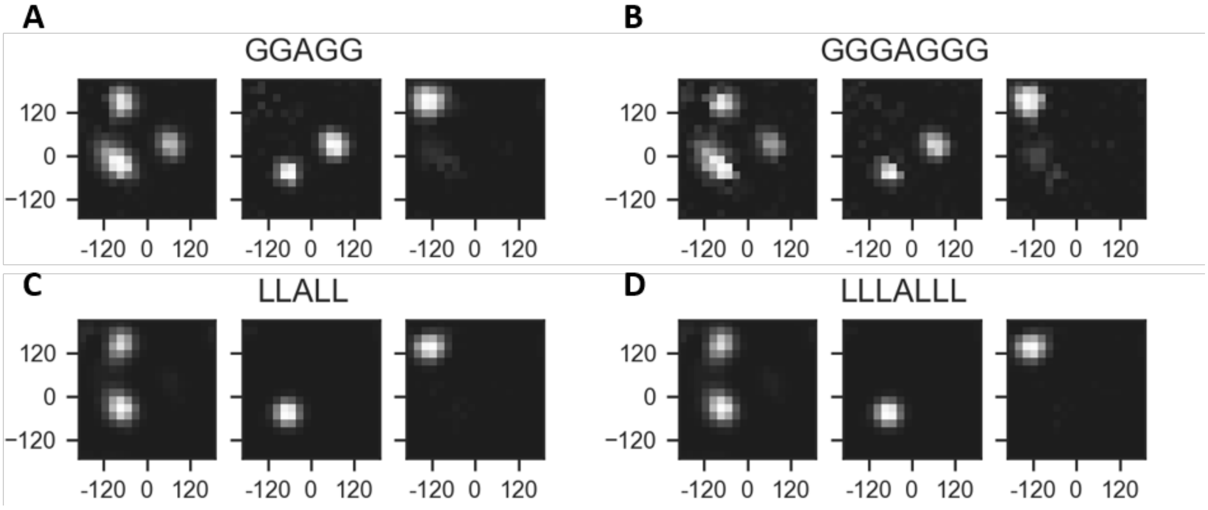

Fig. S3: TALOS-N predictions depend on the amino acid context. Every panel (A-D) comprises predictions of phi/psi maps for Ala of the peak maximum / mixed propensity point (left), a helix-like point (middle) and a sheet-like point (right). Chemical shifts for other residues were propensity- and residue-type corrected as described in the main text. In run B additional glycines were prepended and appended. In runs C and D glycines were artificially replaced (for the TALOS input) with leucines. This experiment demonstrates that TALOS-N returns predictions without the right-handed helical component for a (normal) sequence with (bulkier) sidechains. In case of no sidechains (A and C), the right-handed helical component is lower in the case of a 7-mer, which is probably related to the overall confidence of TALOS prediction.

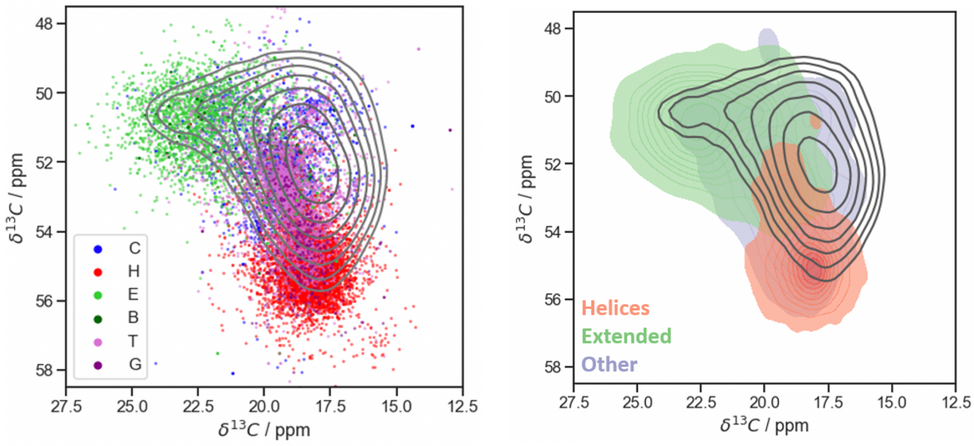

Fig. S4: Derivation of secondary-structural regions depicted as shades in Main Text Figs. 2 and 3. The colored shades were obtained by kernel density estimation, as implemented in Python, by taking into account 96 % of the entry density of the PACSY data base.

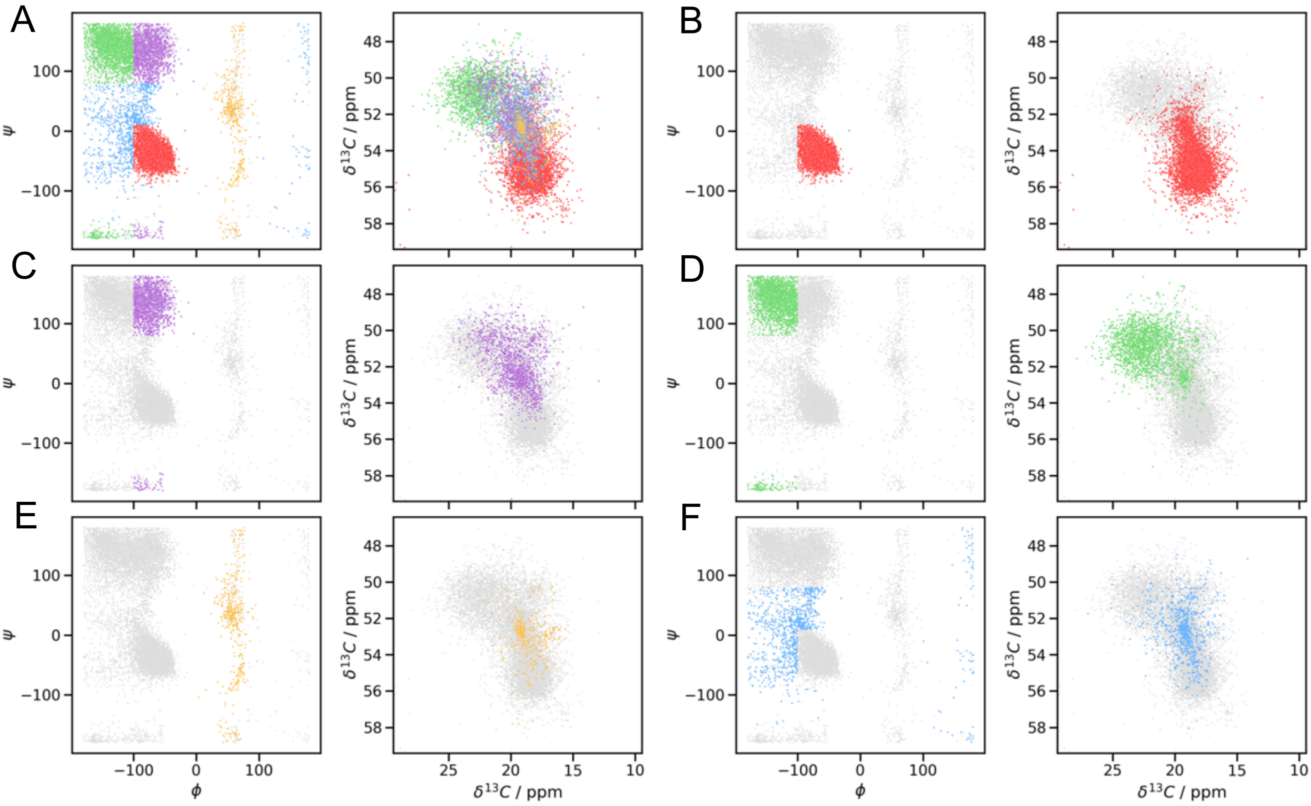

Fig. S5: All alanine entries of PACSY database, colored by dihedral angle combinations. Panel A shows all the entries excluding those classified as random coil ("C"); panels B-F show the five regions separately with all other points from the panel A being grayed out. Note that whereas the overall trends are similar, the correlations between angles and shifts are slightly different for the other 19 amino acids (data not shown).

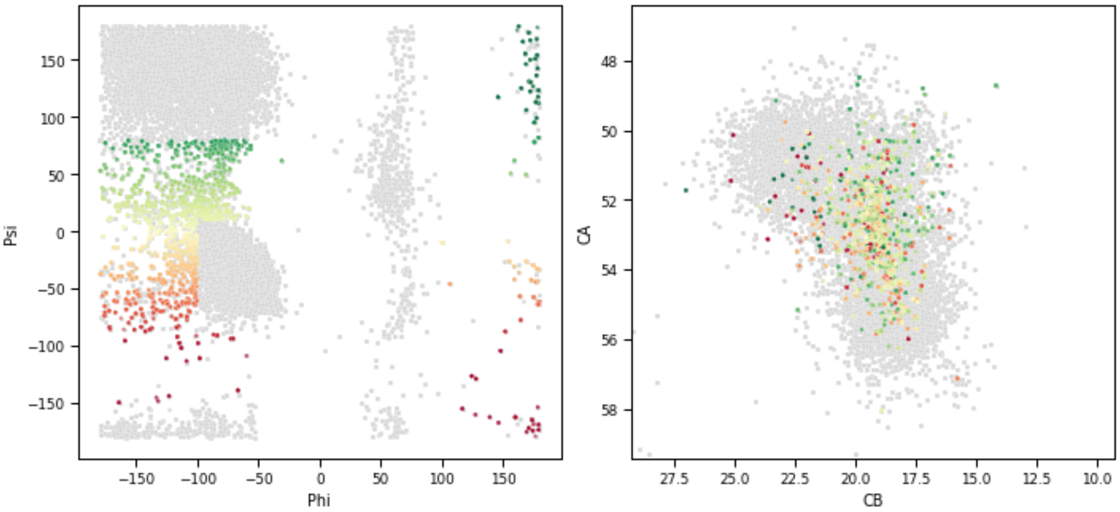

Fig. S6: Correlation of phi/psi angles within the blue category of Fig. S5 with Ca/Cβ carbon chemical shifts.

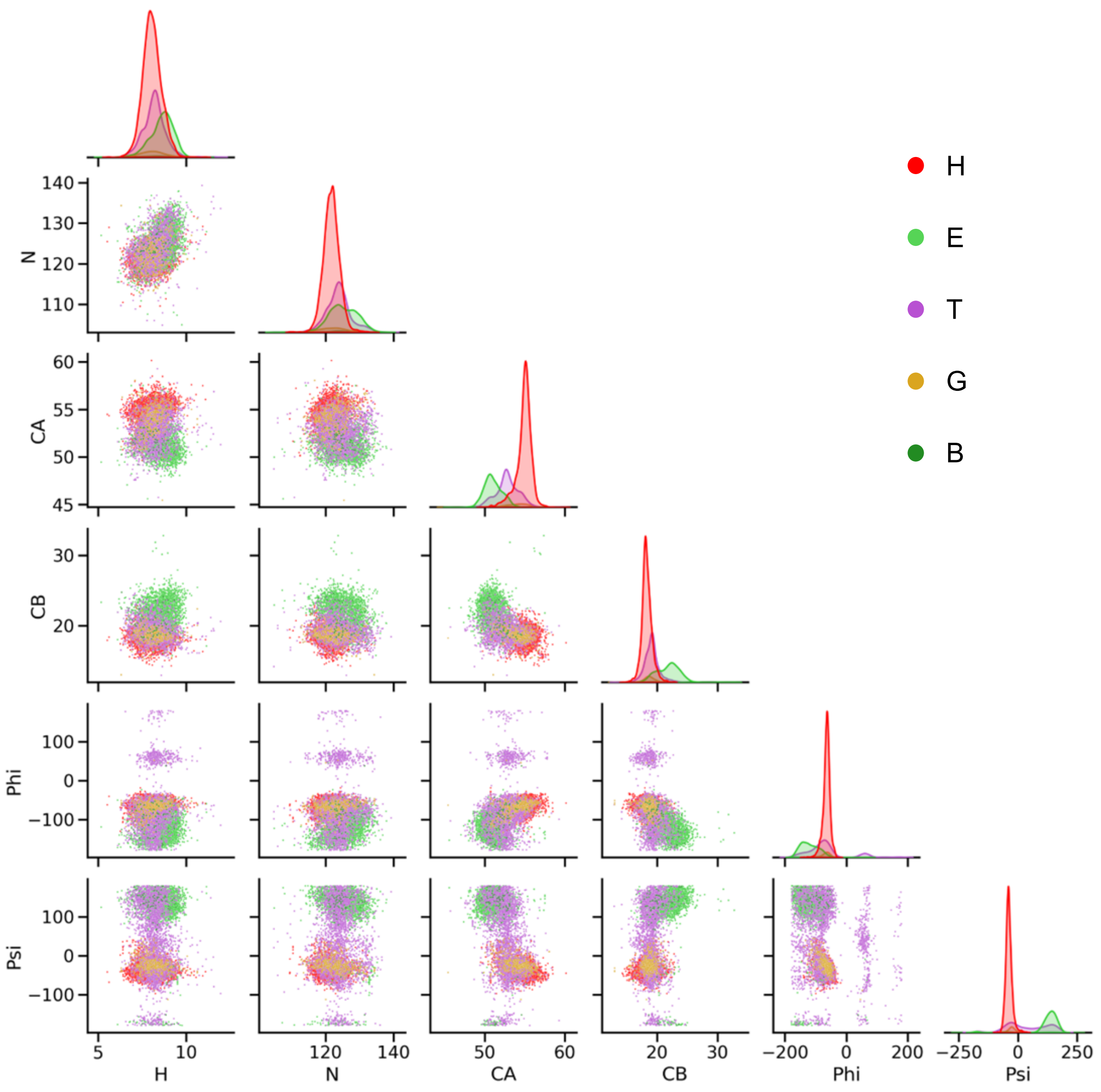

Fig. S7: A pair-wise relations of the four chemical shifts and the two backbone dihedral angles. Points are colored according to the STRIDE classification of the residue: H – α-helix, E – β-sheet, T – turn, B – isolated beta-strand, G – 3-10 helix. Rare classes ("I", "b") as well as random coil ("C") are omitted.

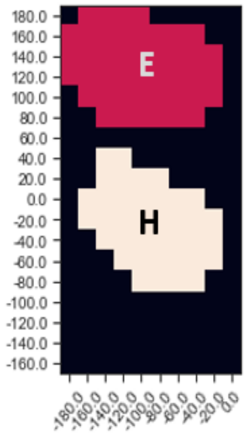

Fig. S8: Regions used in calculation of the *R* score. The red region and tan regions, marked with E and H correspondingly, denote pixels of the 18x10 phi/psi plot that were integrated into E and H parameters.

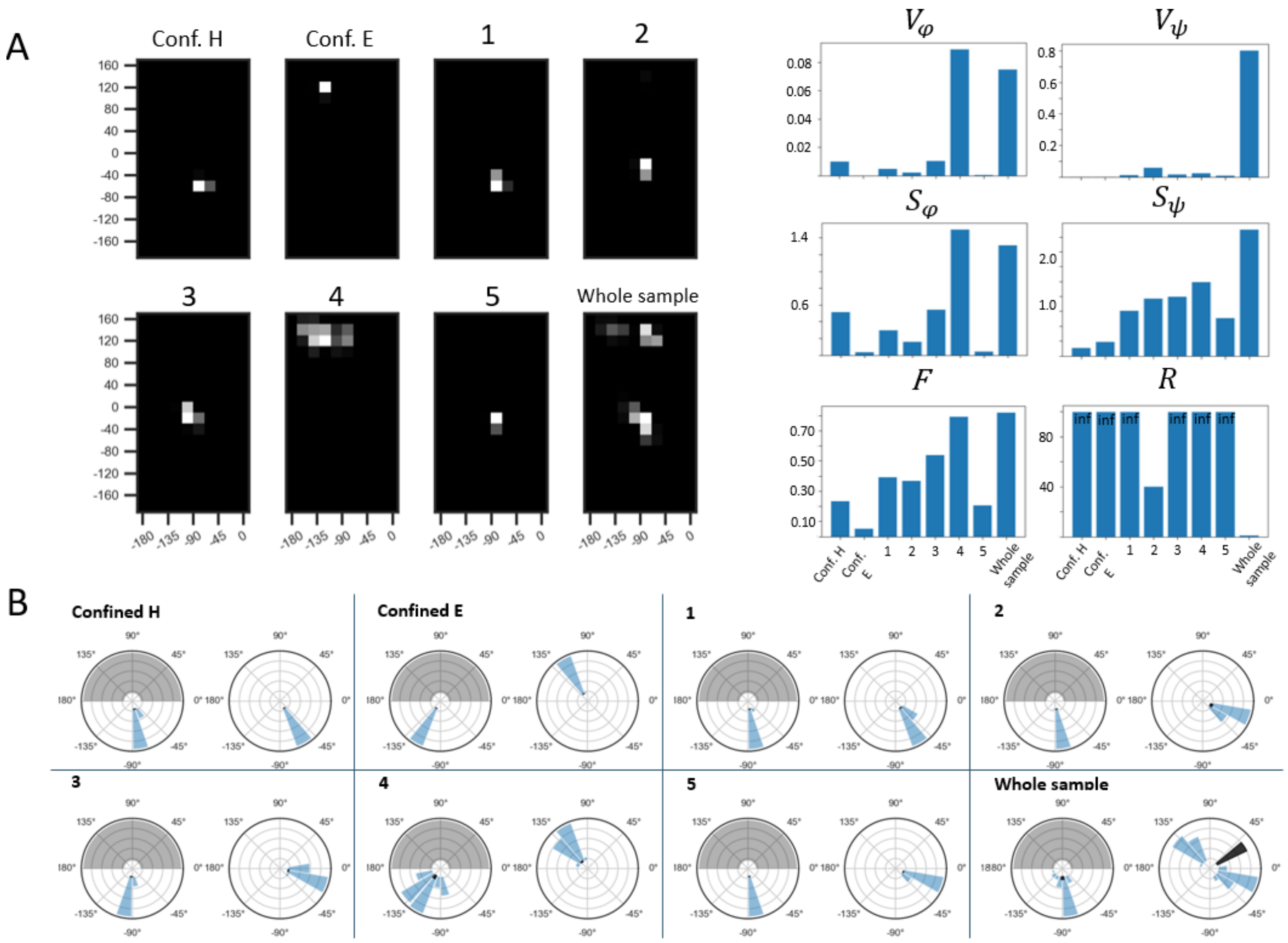

Fig. S9. Tests and reconstruction of conformational distributions based on dihedral-angle predictions with DANGLE. The plots are entirely analogous to those in Main Text Figs. 4, 6B, and 6C. **A)** Folded Ramachandran maps of the test coordinates. Panel "Whole sample" corresponds to the weighted sum of predictions over the whole Ala peak of heterogeneous GGAGG. For generation of pure secondary structure, predictions were made of the 5th Leu in a Leu_{10} chain with the corresponding expected chemical-shifts values (taken from main text reference 46). Grayscale is normalized from 0 (black) to 1 (white, maximum value). **B)** Ramachandran maps from A) in polar coordinates. In each pair, the left plot corresponds to φ and the right one to ψ distributions. The gray area denotes the non-valid φ region for the calculations due to folding (see main text for details). Black vectors point into the *mean direction*, their length is set here to represent the circular variance, not the length of the resulting vector for the distribution. **C)** Representation of different measures of heterogeneity (circular variance V, entropy S, flatness F, and secondary-structure ratio R) for the maps shown in A) as bar plots.

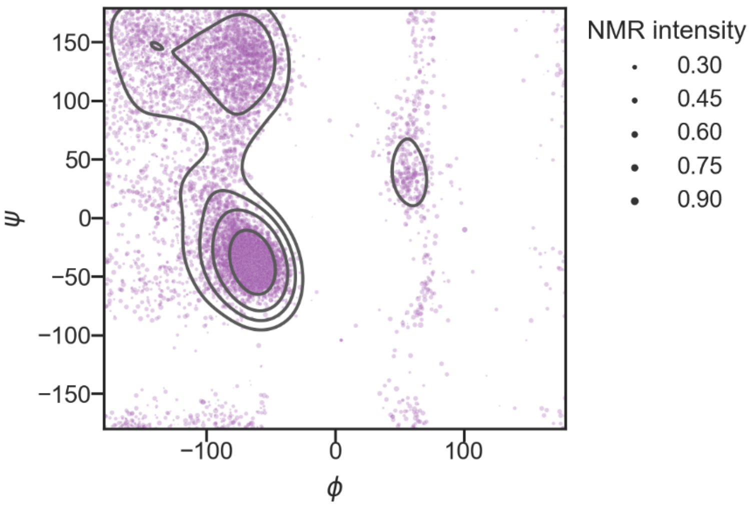

Fig. S10: PACSY entries, included in the volume of the 4D CACB crosspeak in GGAGG HNCACB spectrum. Sizes of the points represent the peak intensity (relative to peak maximum). Contours represent weighted density estimate (starting from 0.15 relative point density and succeeding with the factor of 1.1)

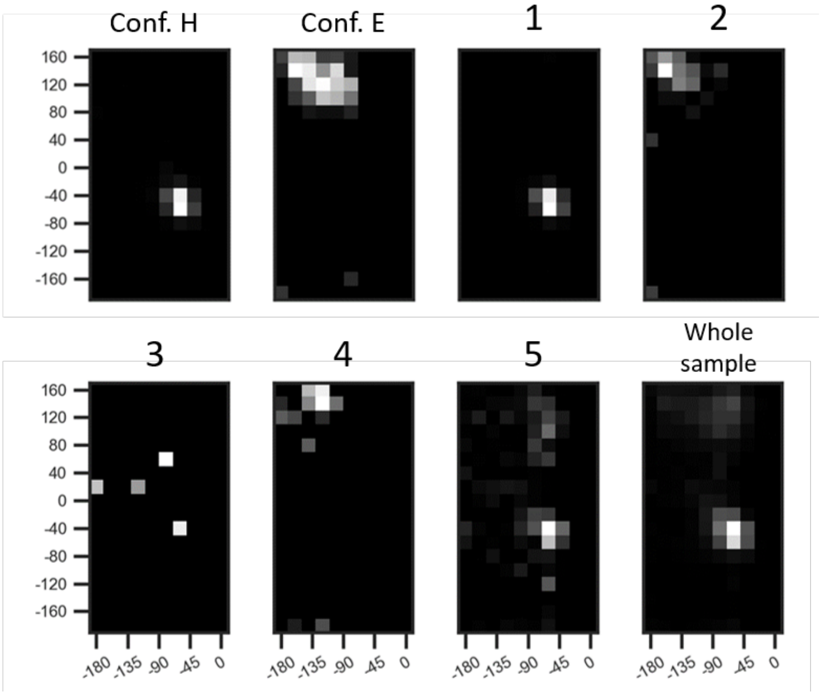

Fig. S11: Folded ϕ/ψ distributions obtained for the distributions of PACSY entries, weighted with the product of the corresponding 4D crosspeak intensity and inverse point density.
